# Telemedicine for Managing Type 1 Diabetes in Children and Adolescents Before and After the COVID-19 Pandemic

**DOI:** 10.3390/jcm13237359

**Published:** 2024-12-03

**Authors:** Federica Fogliazza, Vanessa Sambati, Brunella Iovane, Pietro Lazzeroni, Maria Elisabeth Street, Susanna Esposito

**Affiliations:** 1 Pediatric Clinic, Department of Medicine and Surgery, University of Parma, 43126 Parma, Italy; federica.fogliazza95@gmail.com (F.F.); vanessa.sambati@unipr.it (V.S.); mariaelisabeth.street@unipr.it (M.E.S.); 2 Unit of General Pediatrics and Pediatric Emergency, University Hospital of Parma, 43126 Parma, Italy; biovane@ao.pr.it (B.I.); plazzeroni@ao.pr.it (P.L.)

**Keywords:** telemedicine, type 1 diabetes, pediatric diabetes management, COVID-19, glycemic control

## Abstract

The COVID-19 pandemic has catalyzed the rapid expansion of telemedicine for managing chronic conditions such as type 1 diabetes (T1D) in children and adolescents. This narrative review aims to explore the role of telemedicine in pediatric T1D management by comparing its use before and after the pandemic. We conducted a comprehensive literature review covering studies published between 2000 and 2024, focusing on telemedicine applications in pediatric T1D care. The review includes clinical trials, systematic reviews, and observational studies examining telemedicine’s impact on glycemic control, patient satisfaction, and healthcare delivery. Results reveal that telemedicine has enhanced access to care, improved glycated hemoglobin (HbA1c) levels, and reduced diabetic ketoacidosis and hypoglycemic events. Patients and caregivers expressed high satisfaction, especially when using continuous glucose monitoring and insulin pump technologies integrated with telemedicine platforms. However, challenges such as digital literacy gaps, variability in healthcare provider training, and logistical issues like reimbursement policies persist. The pandemic highlighted the potential of telemedicine to supplement traditional in-person care, showing promise in enhancing patient outcomes and reducing healthcare burdens. Further research is needed to optimize telemedicine models for T1D, addressing barriers to implementation and exploring its long-term cost-effectiveness. This review underscores telemedicine’s evolving role as a complementary approach in managing pediatric T1D, advocating for the development of standardized care protocols to fully integrate digital health solutions into routine clinical practice.

## 1. Introduction

Diabetes is a multifaceted metabolic disorder characterized by chronic hyperglycemia stemming from defects in insulin secretion, insulin action, or both. Various types of diabetes have been identified and classified based on etiopathogenesis [1]. Type 1 diabetes (T1D) is a condition in which the immune system targets and destroys insulin-producing pancreatic cells, resulting in a complete lack of insulin. Consequently, individuals with T1D require progressively higher doses of insulin over time [2,3]. This condition is the most prevalent form of diabetes in children and adolescents, affecting at least 85% of individuals under 20 years of age globally [4].

Continuous glucose monitoring (CGM) has significantly transformed the management of T1D in pediatric populations. These devices utilize a small sensor inserted under the skin to provide near-continuous blood glucose data [2]. By measuring glucose levels in the interstitial fluid, CGMs offer readings every 5 to 15 min, although these may slightly differ from traditional finger-prick blood sugar tests. Real-time CGM devices deliver instant glucose readings, alert users to high or low blood sugar levels and rapid fluctuations, and even predict future trends. All available CGM systems are compatible with smartphone applications, allowing parents and caregivers to monitor glucose levels remotely [3,5]. In managing T1D, children may receive multiple daily insulin injections tailored to their basal and mealtime needs, or they may use insulin pumps, which deliver continuous insulin for more flexible, better-controlled management [6,7].

Effective management of T1D in children and adolescents necessitates a comprehensive approach that addresses glycemic control, nutrition, physical activity, and psychosocial factors. This multifaceted strategy is essential to prevent acute complications and mitigate the risks of cardiovascular and microvascular diseases [8,9,10]. As highlighted by the International Society for Pediatric and Adolescent Diabetes (ISPAD), “Every child has the right to structured, comprehensive education to enable them and their families to manage diabetes effectively” [11]. In this context, telemedicine and new diabetes technologies play a crucial role in enhancing patient outcomes by increasing access to T1D care and health information [12,13]. According to a consensus document by prominent Italian Scientific Societies, a collaborative approach between healthcare professionals offering telemedicine services and young patients with chronic diseases can significantly advance healthcare delivery. Esposito et al. emphasize that telemonitoring empowers patients to manage their conditions independently, facilitates remote monitoring, and promotes earlier hospital discharge, ultimately reducing healthcare resource utilization and lowering the risk of complications [14].

To ensure the success of telemedicine, it is vital that healthcare providers and patients are adequately trained and aware of their roles within the system. This includes addressing the evolving doctor–patient relationship and ensuring that high-quality care persists even in remote settings [15]. However, a notable disadvantage of telemedicine is the digital literacy gap, which particularly affects older individuals and those lacking reliable internet access [14]. Despite these challenges, T1D technology combined with telemedicine has shown promise as a valuable adjunct to traditional care, particularly in pediatric diabetes management, contributing to improved glycated hemoglobin (HbA1c) levels and reduced incidences of diabetic ketoacidosis and severe hypoglycemia [16]. Numerous studies highlight the potential of these technologies to support T1D self-management, especially when physical distance hinders direct care [10,16,17,18,19,20]. Nonetheless, more literature is needed on the growing adoption of virtual consultations and telemedicine despite its increasing use [21]. As T1D prevalence rises, the development of efficient, user-friendly platforms that facilitate robust communication between patients and healthcare providers becomes even more critical [22].

It is important to consider the dynamic nature of childhood, where changes in insulin sensitivity due to physical growth, sexual maturation, self-care abilities, and supervision in school and childcare environments must be addressed [11]. Bassi et al. demonstrated that online educational programs for schools improved theoretical knowledge and confidence in managing the daily needs and emergencies of students with T1D [23].

The COVID-19 pandemic led to a rapid global expansion of telemedicine to deliver healthcare services for chronic conditions [24,25]. Although digital transformation in healthcare was already underway, the pandemic accelerated this shift, with many young patients with diabetes and their families expressing high satisfaction with virtual care [26]. Tornese et al. reported improvements in T1D management across various Italian pediatric diabetes centers through telemedicine, particularly for patients using insulin pumps, who benefitted from enhanced communication with their healthcare teams via data-sharing platforms [27].

This narrative review aims to explore the significance of telemedicine in the management of children and adolescents with T1D, comparing its application before and after the COVID-19 pandemic.

## 2. Methods

We conducted a comprehensive literature review by searching the Medline database via the PubMed interface (https://pubmed.ncbi.nlm.nih.gov/; accessed on 1 September 2024) and Google Scholar (https://scholar.google.com/; accessed on 1 September 2024). Our search focused on articles published between January 2000 and July 2024. The inclusion criteria prioritized systematic reviews (SRs), meta-analyses (MAs), randomized controlled trials (RCTs), and clinical trials (CTs) involving patients with T1D and their families who used innovative applications and web-based telemedicine systems.

The search strategy was designed to ensure a broad and thorough review of relevant studies. We used keywords such as “type 1 diabetes”, “telemedicine”, “remote care”, “virtual consultations”, “CGM”, “insulin pump management”, and “COVID-19 and diabetes care” to capture a wide range of research. Boolean operators (AND, OR) were used to combine terms effectively, and filters were applied to refine the results of studies involving human subjects and published in English.

Given the limited number of sources specifically focusing on pediatric populations, we included studies with both pediatric and adult T1D patients to enhance the comprehensiveness of our findings. This allowed us to extract insights relevant to the overall management of T1D through telemedicine, regardless of age group. Additionally, studies that focused on T1D management during the COVID-19 pandemic were included, even if telemedicine was not the primary subject, as the pandemic played a pivotal role in accelerating the adoption of digital health solutions.

We also imposed specific eligibility and exclusion criteria. Studies were eligible if they explicitly involved the use of telemedicine to deliver healthcare to patients with T1D, including remote glucose monitoring, insulin management, or education. We excluded studies published before 2000 to focus on more recent technological advancements. Additionally, studies not published in English were excluded to ensure accessibility and consistency in reviewing the available data.

Finally, data from the selected studies were extracted systematically. This included study design, population characteristics, type of intervention (e.g., telemedicine platform or digital tool), outcome measures (e.g., HbA1c levels, incidence of diabetic ketoacidosis, patient satisfaction), and study findings related to the effectiveness of telemedicine in managing T1D. Where possible, we cross-referenced findings from multiple studies to identify consistent trends and discrepancies in the outcomes reported.

## 3. Results

Following the initial search, a total of 32 articles were selected for review: 13 were published prior to the COVID-19 pandemic, and 19 after the pandemic began. Information on the study period (i.e., pre-pandemic or post-pandemic) was found within the article. These articles have been organized into two tables (Table 1 [28,29,30,31,32,33,34,35,36,37,38,39,40] for pre-pandemic studies (n = 13) and Table 2 [9,21,26,41,42,43,44,45,46,47,48,49,50,51,52,53,54,55,56] for post-pandemic studies (n = 19), with each entry categorized by title, study location, study design, population, study aim, summary of results, and conclusions.

Of the selected studies, 34% (11 studies) were conducted in Europe, 50% (16 studies) in the United States, and 12% (4 studies) in Asia and the Middle East. One observational multicenter study spanned Europe, America, and Asia, representing 3% of the total studies. Most of the research focused on evaluating the effectiveness and reliability of telemedicine systems, such as applications, online platforms, virtual groups, and video-mediated support for T1D patients and their families. The outcomes assessed included glycemic control, represented by clinical measures such as HbA1c levels, physical and psychological distress in pediatric, adolescent, and adult patients, family burden, patient and healthcare provider satisfaction, and the integration of in-person and telemedicine care models.

The most frequently analyzed primary outcome was metabolic control, specifically changes in glycated hemoglobin (HbA1c). Across studies, telemedicine interventions were generally associated with moderate reductions in HbA1c or similar outcomes compared to in-person visits, indicating its potential as an effective tool for managing T1D [17]. Another key outcome related to quality of life was often measured through standardized scores that addressed concerns such as fear of hypoglycemia and the psychological toll of managing a chronic condition. Telemedicine systems, especially those that facilitated direct connections between patients, families, and healthcare providers, consistently demonstrated improvements in patient quality of life, particularly for adolescents. Additionally, the use of virtual platforms led to a significant reduction in missed work or school hours for both patients and their families, highlighting its practical advantages [31,42].

Healthcare provider satisfaction was another secondary outcome, though less frequently studied, likely due to the variability in assessment methods across different care centers. Satisfaction among healthcare staff was often linked to the ease of integrating telemedicine into existing care models, though this outcome was less standardized and more dependent on the specific characteristics of each care setting [10].

Overall, telemedicine was found to yield results that were comparable to, or better than, traditional in-person visits at diabetes care centers. However, several challenges were noted. First, telemedicine must be tailored to individual patients, as highlighted in Rachmiel et al.’s observational multicenter study, which indicated that telemedicine was not suitable for all patients [49]. Although telemedicine offers the potential to bridge gaps between patients and healthcare providers, it also requires access to reliable electronic tools—a barrier for some populations [38,51]. Additionally, issues surrounding reimbursement and the time healthcare providers must allocate to telemedicine appointments remain unresolved, with these challenges being influenced by the structure of local healthcare systems [13,32,36,39].

In addition to clinical studies, we also evaluated ten reviews, including systematic reviews and meta-analyses. Three of these reviews were conducted before the pandemic and primarily focused on exploring the potential of telemedicine as an alternative to traditional in-person care, raising concerns about its feasibility and effectiveness [28,33,36]. The seven reviews conducted post-pandemic, however, were more optimistic, emphasizing the proven utility of telemedicine during extraordinary circumstances and advocating for its broader adoption in clinical practice [9,19,44,45,51,52,53].

Despite the positive outcomes reported, all studies consistently underscored the need for further research to develop a more standardized care model that fully integrates telemedicine and digital support tools into diabetes management. The growing use of these technologies, particularly during the pandemic, has revealed their potential but also highlighted gaps in understanding how to optimize their use across different populations and healthcare systems.

## 4. Discussion

To our knowledge, this is the first review to specifically examine the evolution of telemedicine in T1D care in relation to the pandemic, highlighting how its use and perception have shifted over time. By providing this temporal comparison, our work contributes to the growing body of literature that underscores the potential of telemedicine to transform chronic disease management, particularly in pediatric populations.

The literature consistently demonstrates that telemedicine has evolved into an indispensable tool in clinical practice, particularly in the management of chronic conditions like T1D. Our review highlights a significant increase in the attention given to telemedicine for diabetic patients, especially during the COVID-19 pandemic. The pandemic created a unique, nearly universal condition in which geo-graphic limitations and disparities in healthcare systems were, to some extent, equalized. The necessity for social distancing compelled all healthcare systems, regardless of their pre-pandemic telemedicine infrastructure, to adopt and expand telehealth solutions. This forced adoption fostered the rapid development and integration of telemedicine, even in regions where its use had been limited prior to the pandemic.

In the pre-COVID era, relatively few clinical trials had been conducted to assess the efficacy of telemedicine in managing chronic pediatric conditions, including T1D [35]. As a result, there was insufficient evidence to draw firm conclusions about its broader applicability. However, in the years following the pandemic, the growing reliance on telemedicine has led to a shift in research priorities. While clinical trials continue to be important, the current landscape now demands more systematic reviews that can synthesize data from diverse populations and assess the broad benefits of telemedicine. These reviews provide a more comprehensive understanding, helping to mitigate biases introduced by individual studies or specific telemedicine platforms.

The sophistication of telemedicine platforms has markedly increased, offering near-instantaneous support and enabling direct access to multidisciplinary teams. Before the pandemic, telemedicine was largely viewed as an alternative or supplementary option to traditional care models. Now, many studies position telemedicine as a central element of routine clinical care, particularly for chronic disease management. Importantly, telemedicine is not considered a replacement for in-person visits but rather a complement that enhances the overall care experience by providing continuous, flexible support.

Our findings also confirm that telemedicine has a positive impact on glycemic control, as evidenced by improvements in HbA1c levels reported in numerous studies. However, additional research is needed to fully understand the broader clinical impacts, particularly concerning cardiovascular risks and metabolic syndrome, which are critical concerns for diabetic patients [33,51]. Further exploration of these outcomes could enhance the comprehensive understanding of telemedicine’s role in managing these aspects of diabetes care.

Telemedicine offers unique opportunities to address the specific developmental and psychosocial needs of children and adolescents with T1D [57,58,59]. This age group often requires tailored support to manage the emotional and behavioral challenges associated with diabetes, such as adherence to treatment regimens, dealing with peer dynamics, and fostering independence in self-care. Telehealth enables frequent, flexible interactions that can be more accessible and less intimidating than traditional clinic visits, allowing providers to build rapport and offer guidance in real time. Additionally, the integration of virtual platforms with digital health tools empowers young patients to actively participate in their care while providing families and caregivers with reassurance and support.

The training of healthcare providers for managing T1D via telehealth is a critical component of modern diabetes care [57,58,59]. Effective telehealth practices require providers to be proficient not only in the technical aspects of virtual platforms but also in communication strategies tailored to remote settings. This training should emphasize the integration of telehealth as a complementary tool rather than a replacement for in-person visits. Telehealth allows for more frequent touchpoints, real-time data sharing, and timely interventions, which are essential for managing T1D. However, certain aspects of care, such as physical examinations and advanced diagnostics, remain reliant on face-to-face interactions. By equipping providers with skills to balance telehealth and in-person care, healthcare systems can optimize outcomes while ensuring comprehensive and personalized management of T1D [57,58,59].

Our review has certain limitations. First, we conducted a descriptive rather than a systematic analysis of the sources, which may introduce selection bias. This approach limits our ability to definitively evaluate the quality and generalizability of the studies included. However, while a systematic review would have provided a stronger design with more comprehensive and structured evidence synthesis, the search methods employed in this narrative review appear reasonable and adequately support the scope of the study. Additionally, we reviewed a mix of clinical trials and studies of varying hierarchical importance, which may affect the robustness of our conclusions. However, this broader inclusion could also be seen as a strength, as our goal was not solely to assess the effectiveness of telemedicine but to provide a comparative analysis of its role in the pre- and post-pandemic periods.

## 5. Conclusions

The growing body of evidence confirms that telemedicine is a valuable tool for managing chronic illnesses such as diabetes, particularly in pediatric and adolescent populations. Clinically, telemedicine offers substantial benefits, including improved glycemic control, enhanced quality of life, and increased convenience for both patients and healthcare providers. However, it is crucial to recognize that telemedicine should complement, not replace, traditional in-person medical, nursing, and psychological care. The integration of telehealth into routine diabetes management must be carefully structured to ensure it enhances, rather than de-tracts from, the holistic care patients receive.

Future research should prioritize studies with larger, more representative samples that include diverse populations and various types of diabetes. In particular, there is a pressing need for studies that examine the differential effects of telemedicine interventions based on patient age, the chronicity of the disease, and the involvement of caregivers in pediatric patients’ daily lives. Pediatric-focused research remains limited, and expanding this area with more extensive, targeted trials is critical to fully understanding the nuances of telemedicine in young diabetic populations.

Another important clinical implication is the shift towards personalized, patient-centered care. Telemedicine should align with this approach, providing tailored interventions while maintaining a baseline of standardized care that ensures equitable access across different healthcare systems. Future research must also address the logistical challenges of telemedicine, such as the time demands placed on healthcare providers and the need for clear reimbursement models. The economic regulation of telemedicine, including cost-effectiveness and sustainability, is a critical area for future exploration to ensure its long-term viability in clinical practice.

Once the effectiveness of telemedicine is firmly established, subsequent studies should delve deeper into specific applications, such as the use of CGM systems in pediatric populations. Research should focus on optimizing visit times, assessing associated costs, and identifying barriers to the implementation of telemedicine in routine clinical practice. These studies will be essential for refining telemedicine models to ensure they are both effective and practical in the long term. Addressing these gaps will help solidify telemedicine’s role in the future of T1D care, particularly for vulnerable and high-need populations like children and adolescents.

## Figures and Tables

**Table 1:** Studies on the use of telemedicine for the management of children and adolescents with type 1 diabetes (T1D) published before the COVID-19 pandemic.

Study	Placement	Study Design	Population	Study Aim	Summary of Results	Conclusions
Döğer et al. [28]	Gazi University Faculty of Medicine, Ankara, Turkey	Clinical trial	Diabetic children aged between 2 and 18 years. In the study group, 14 (17.1%) were using pumps, while 59 (72.0%) counted carbohydrates. Of the participants, 78 (95.1%) attended their check-up visits regularly.	To assess the effects of counselling services offered to diabetics by the diabetes team via communication networks (internet, phone) on diabetes control.	HbA1c values after six months were significantly lower in patients/parents calling frequently (*p* < 0.001) compared with HbA1c values recorded at the beginning of the study. The frequency of use of WhatsApp (28.0%) was higher than the other means of communication.	An increase in the frequency of counselling by the diabetes team led to improved blood glucose control in T1DM patients. A telehealth system is useful for early detection of the need for changes in treatment and for intervention. It also promoted better self-care.
Crossen et al. [29]	Department of Pediatrics, University of California, Davis Health System, Sacramento, California Department of Public Health Sciences, University of California, Davis, Davis, California, USA	Non-randomized clinical trial	Children aged 1–17 years with diagnoses of T1D >3 months prior, HbA1c level ≥8% (64 mmol/mol), access to the internet, and technologies (insulin pump or CGM).	To evaluate the effects of home-based video visits in T1D pediatric patients with suboptimal glycaemic control.	The study cohort demonstrated significant improvement in mean HbA1c (from 10.8% at baseline to 10% at 10–12 months, and participants who completed 12 months of video visits declined to 9.6%).	Home-based video visits are a feasible supplement to in-person care for children with T1D and suboptimal glycaemic control and can improve both HbA1c level and adherence to medical care.
Los et al. [30]	Oregon Health & Science University, Portland, OR, USA	Review	The available resources, patterns of use in transition-age youth, and opportunities to advance technology use in transitioning patients with T1D from pediatric to adult care.	To review the use of technology for patients with T1D transitioning from pediatric to adult care, day-to-day management of diabetes, and the use of technology in health care delivery and patient-provider communication (telemedicine) in this population.	The potential role for diabetes technologies: online assessment tools; enhancement in health literacy and disease management skills; behavioral health interventions via video conferences; pump and CGM devices to facilitate real-time decision-making; integrated electronic medical record platforms enabling a timely exchange of data between patient and provider.	Evidence points to a more successful transfer of care during the transition age in patients using technology for insulin delivery and glucose monitoring. Patterns and barriers to telehealth utilization in transition-age youth with T1D should be studied more systematically. Integrated platforms that give access to multiple users should be developed.
Frielitz et al. [31]	University Medical Center, Campus Luebeck, Germany	“ViDiKi” (Virtual Diabetes Outpatient Clinic for Children and Youth). Multicentre Controlled trial.	240 participants aged between 1 year and 16 years using a CGM with multiple daily injections (or insulin pump therapy. The controlled part of the study analyzes the effect of six months of additional telemedical care compared with regular care only.	To examine if monthly telemedical consultations, in addition to regular care, will improve glycemic control and psychosocial outcomes. The sample size is designed to detect a between-group difference of 0.5% in HbA1c change at six months.	Mean changes in glycated hemoglobin (HbA1c), from baseline to 6 months, are compared between the two groups with a linear regression, adjusted for baseline HbA1c. Adolescents and their caretakers complete the Diabetes Treatment Satisfaction Questionnaire, status version. Responses on treatment satisfaction and perceived metabolic control are summarized and given on a seven-point scale. Higher scores indicate greater satisfaction with diabetes treatment.	ViDiKi provides new evidence of how telemedical care may impact the metabolic control and day-to-day living of young patients with T1D and their families and gives insight into the experiences of HCPs with using telemedicine, as well as the costs of this new model of diabetes care.
Frøisland et al. [32]	Lillehammer University College, Lillehammer; University Hospital of North Norway, Tromsø, Norway	Clinical trial	12 adolescents aged between 13 and 19 years in Eastern Norway. The participants were diagnosed with T1D at least 1 year prior to the start of the study, and they had a current HbA1c of less than 10.0%.	To affect understanding of carbohydrate counting and to facilitate doctor–adolescent communication about daily treatment, evaluate the effect of the designed tool regarding empowerment, self-efficacy, and self-treatment.	Adolescents found both mobile applications useful in supporting their diabetes self-management. Glycemic control, as measured by HbA1c, improved in seven out of eleven participants completing the study.	Implementing a visualization tool is an important contribution that helps young people understand the basics of diabetes and empowers them to define their treatment challenges.
Giani et al. [33]	Harvard Medical School, Boston, MA, USA	Observational study	70 young persons with T1D, 1–22 years of age. Age and duration were similar among the 54 patients who completed 12 months of follow-up, and these 54 served as the basis for the satisfaction analyses.	To assess HbA1c outcomes and visit frequency over 1 year of follow-up care using telemedicine, these outcomes will be compared with historical outcomes in the year prior to the implementation of telemedicine among the same patients.	The mean A1c did not change significantly from baseline. Telemedicine visits were non-inferior to face-to-face care for pediatric patients from rural areas. Visit frequency was greater in the year with telemedicine compared with the prior year. Participants reported significantly fewer missed work/school hours. Satisfaction was high with telemedicine.	Telemedicine is becoming an increasingly important alternative to delivering healthcare, and it has the potential to address disparities related to access by overcoming barriers of distance, time, and possibly expense. Challenges remain related to reimbursement, identifying which patients may benefit most, and understanding the optimal frequency of telemedicine visits as a replacement for in-person encounters.
Tonyushkina et al. [34]	Baystate Children’s Hospital, Springfield, MA, USA	Prospective pilot study	750 children and young adults with T1D as part of an academic tertiary health care centre. All visits were followed by a written communication (e-mailed securely), including insulin adjustments and recommendations regarding diabetes self-management. One or both parents participated in televisits with children and adolescents.	To determine the feasibility and acceptance of a yearlong intervention substituting three of four yearly recommended clinic visits with direct patient–physician video calls via Vidyo Inc.	HbA1c did not change significantly over the year. All participants expressed high satisfaction with care and would like to continue with the televisits. Males might be more likely to enroll and engage in diabetes decision-making via a video conference.	Patient–physician video conferencing is feasible and highly accepted by motivated and tech-savvy patients with T1D and their families to substitute for half of the recommended clinic visits with coordination and tech support to ensure timely glucose data upload. Further controlled and randomized studies of diabetes televisits will help build a path for policy changes and insurance reimbursement.
Chapman et al. [35]	Monash University Malaysia, Bandar Sunway, Malaysia; UCSI University, Kuala Lumpur, Malaysia	A systematic review and meta-analysis of randomized controlled studies	A total of 38 studies described in 41 articles were identified. Studies with longer duration (>6 months) who had recruited patients with a higher baseline HbA1c (≥9%) were associated with larger effects.	To synthesize evidence and quantify the effectiveness of telemedicine interventions for the management of glycemic and clinical outcomes in T1D patients relative to comparator conditions.	Absolute change in HbA1c from baseline to follow-up assessment. Positive effects on glycemic control were noted in studies examining telemedicine, with a mean reduction of 0.18% at the end of the intervention. Telemedicine interventions with individualized assessments, audits with feedback, and skill-building were also more effective in improving glycemic control. No benefits were observed in terms of blood pressure, lipids, weight, quality of life, or adverse events.	There is insufficient evidence to support telemedicine use for glycemic control and other clinically relevant outcome among patients with T1D.
Lehmkuhl et al. [36]	Nationwide Children’s Hospital, Columbus, Ohio; University of Florida, Gainesville, Florida, USA	Randomized clinical trial.	32 youths (23 female) between the ages of 9 and 17 y. Inclusion criteria were diagnosis of T1D for at least six months, an HbA1c greater than 9%, and the availability of a caregiver to accompany a participant to assessments.	The present study expands the literature in this area by presenting results from a pilot trial of the first randomized waitlist-controlled trial of Telehealth Behavioral Therapy (TBT) for youths with poor glycemic control.	Mean HbA1c scores reduced from 10.2% to 9.8%. For the diabetes family behavior scale (DFBS) and the diabetes family behavior checklist (DFBC), the time per group interaction was significant. For the diabetes family responsibility questionnaire (DFRQ), there was no effect of group membership, and the interaction was not significant.	TBT improves access to knowledgeable providers and results in a clinically significant improvement in glycaemic control. Despite some youths experiencing an increase in unsupportive parental behaviors, TBT is a promising method of service delivery that warrants further investigation.
Freeman et al. [37]	Oregon Health & Science University, Portland, Oregon, USA	Randomized controlled Clinical trial	71 adolescents with poorly controlled T1D (hemoglobin A1c ≥9%) and one of their caregivers received up to 10 sessions of a family-based behavioral health intervention previously shown to improve adherence to diabetes regimens and family functioning; 32 were randomized to the Skype condition.	To compare the therapeutic alliance when behavioral health care was delivered to youth with poorly controlled T1D and their caregivers in-clinic with the same services delivered via internet-based video conferencing (i.e., Skype™).	No significant differences in WAI (Working Alliance Inventory) scores across those receiving BFST-D via Skype versus in-clinic.	Behavioral health can be delivered to youth with T1D via internet-based video conferencing without significantly impacting the therapeutic relationship. For those adolescents with T1D who require specialized behavioral health care that targets T1D management, internet-based teleconferencing represents a viable alternative to clinic-based care.
Edwards et al. [38]	School of Social Sciences, Bangor University, Bangor, UK	Mixed-method systematic review	66 studies were included in the review.	To determine the effectiveness of interventions across all outcomes conducted with children, young people, and school personnel to optimize T1D care and management in educational settings, identify the barriers and facilitators to achieving optimal T1D	Telemedicine in school was effective for individual case management. Most educational interventions to increase the knowledge and confidence of children or school staff had significant short-term effects, but longer follow-up is required.	Telemedicine between healthcare providers and schools, as well as school nurse support for children, is effective in specific contexts, but not all education systems employ onsite nurses. More innovative and sustainable solutions and robust evaluations are required.
Ellis et al. [39]	Carman and Ann Adams Department of Pediatrics, Wayne State University, Detroit; Pediatric Prevention Research Center, Detroit; Medical University of South Carolina, Charleston, SC, USA	Randomized controlled trial	146 adolescents with type 1 or 2 diabetes and their families from 2006 and 2010 and with a current HbA1c of 8% or higher and a mean. Data were collected at baseline, 7 months (treatment termination), and 12 months (6 months follow-up).	To determine whether MST, an intensive, home-based, tailored family treatment, was superior to weekly telephone support for improving regimen adherence and metabolic control among adolescents with chronic poor metabolic control.	Adolescents receiving MST had significantly improved metabolic control at 7 (1.01% decrease) and 12 months (0.74% decrease) compared to adolescents in telephone support. Parents of adolescents receiving MST reported significant improvements in adolescent adherence.	MST improved health outcomes among adolescents with chronic poor metabolic control when compared to telephone support. Home-based approaches may provide a viable means to improve access to behavioral interventions for such youth.
Whittemore et al. [40]	USA	Secondary analysis of data from a clinical trial evaluating the effect of an internet coping skills training program (TEENCOPE) compared to an internet diabetes health education program (Managing Diabetes) for youth with type 1 diabetes.	Youth with T1D from four sites in the United States were invited to participate (N = 510), and 320 eligible youth consented.	To compare the demographic and clinical characteristics of youth with T1D in terms of recruitment, participation, and satisfaction with two eHealth psychoeducational programs.	Significant differences in recruitment rates by income and race/ethnicity, such as black, Hispanic, or mixed race/ethnicity, and low-income youth were more likely to refuse passively than white and higher-income youth, who were more likely to enroll.	Results indicate that black, Hispanic, or mixed race/ethnicity youth and low-income youth with T1D are less likely to enroll in internet-based research than white and higher-income youth. Creative recruitment approaches are needed.

BFST-D: Blood Flow Stimulation Therapy—Diabetic; CGM: Continuous Glucose Monitoring; HbA1c: glycated hemoglobin; MST: multisystemic therapy.

**Table 2:** Studies on the use of telemedicine for the management of children and adolescents with type 1 diabetes (T1D) published after the COVID-19 pandemic.

Study	Placement	Study Design	Population	Study Aim	Summary of Results	Conclusions
Ballesta et al. [41]	Endocrinology and Nutrition, Consorci Sanitari de l’Alt Penedès Garraf, Vilafranca del Penedès, Spain, Endocrinology and Nutrition, Hospital del Mar, Barcelona, Spain, Department of Medicine, Universitat Autònoma de Barcelona, Universitari Mar, Barcelona, Spain	Randomized controlled, open-label, parallel- arm study among adults with T1D	Persons over 18 years with T1D of at least 6 months duration, with internet access, mobile phone, and trained to use the SocialDiabetes^®^ App.	To compare the change in HbA1c at 6 months in T1D care in a rural area between telehealth and in-person visits.	No significant differences in HbA1c between groups were found.	The telehealth model is comparable to in-person visits regarding HbA1c levels at the 6-month follow-up, with significant improvement in some glucose metrics and health-related quality of life. Further studies are necessary to evaluate a more efficient timing of telehealth visits.
Hilliard et al. [42]	Department of Pediatrics, Baylor College of Medicine and Texas Children’s Hospital, Houston, Texas, USA	Pilot Feasibility and Acceptability Study	84 Enrolled families, 80 randomized (55 T1D intervention, 25 usual care control)	To evaluate the feasibility and acceptability of a pilot behavioral intervention delivered to parents of adolescents with T1D via a mobile-friendly web app.	The Type 1 Doing Well Behavioral mHealth intervention for parents of adolescents with T1D is feasible and acceptable. It may pose as an engaging component of future family-based interventions.	Type 1 Doing Well was feasible to deliver and highly acceptable and engaging for parents of adolescents with T1D. It may have a larger impact on behavioral or clinical outcomes as part of a multi-component intervention protocol.
Marker et al. [43]	Clinical Child Psychology Program, University of Kansas, Lawrence, Kansas, USA	Clinical trial	43 enrolled families of young children with T1D ages of 1–6 years, diagnosed with T1D for at least 6 months). 36 families completed the Reducing Emotional Distress for Childhood Hypoglycemia in Parents (REDCHiP) intervention.	To present the trial design, feasibility, and acceptability of a new intervention aimed at Reducing Emotional Distress for Childhood Hypoglycemia in Parents (REDCHiP) of young children with T1D.	Study attrition was 21%, including long-term follow-up (16% before or during the treatment phase). On average, parents attended 94% of intervention sessions, and fidelity to the treatment manual was 89%. Intervention completers reported high satisfaction with the treatment groups (89% average satisfaction rating).	The REDCHiP intervention demonstrated initial feasibility and acceptability.
Holtz et al. [44]	Michigan State University, East Lansing, Clamson, Detroit, USA	Pilot study	30 adolescent-parent pairs.	To determine the initial efficacy of the MyT1DHero intervention in improving diabetes outcomes in adolescents, specifically the HbA_1c_ levels, diabetes care adherence, and quality of life, and the adolescents’ overall satisfaction with this intervention	Higher use of the mobile app was associated with more improvement in HbA_1c_ levels (F_1,20_ = 9.74, *p* < 0.005; R^2^ = 0.33). Overall, the adolescents were satisfied with the app intervention.	In a 12-week pilot study of the mobile app intervention designed to facilitate parent-adolescent communication for improving diabetes outcomes, significant benefits were demonstrated in self-care adherence and quality of life.
Garcia et al. [45]	Children’s Hospital Los Angeles, Los Angeles, CA, USA. University of Southern California, Los Angeles, CA, USA. Rutgers Robert Wood Johnson Medical School, New Brunswick, NJ, USA	Randomized controlled trial	68 Adolescents and Young Adults (AYAs) with T1D.	Secondary analysis that evaluates diabetes health-related outcomes associated with higher telehealth usage (>50% of visits via telehealth) compared to higher usage of in-person care (>50% of in-person visits).	68 AYAs participants, individuals (n = 39, 57%) who attended most (>50%) study visits by telehealth completed more diabetes care visits (3.3 visits) than those (n = 29, 43%) who primarily attended visits in-person (2.5 visits; *p* = 0.007). AYAs who primarily attended visits via telehealth maintained stable physician-related distress, while those who attended more in-person visits reported increases in physician-related distress (*p* = 0.03).	Greater usage of telehealth improved AYA engagement with their care, resulting in increased clinic attendance and reduced physician-related diabetes distress. A person-centered care model delivered via telehealth effectively meets the needs of AYAs with T1D.
D’Annunzio et al. [46].	Genoa, Verona, Ancona, Novara, Cremona, Rome, Italy	Review	Search in guideline databases, Medline and Embase, and Diabetes Societies websites until 21 May 2020, for guidelines and recommendations on T1D mellitus management during the COVID-19 pandemic.	To review the impact of COVID-19 pandemia in children and adolescents with T1D mellitus, to analyze the clinical characteristics of the infection, and to propose clinical practice recommendations from the Italian Society for Pediatric Endocrinology and Diabetology (ISPED).	COVID-19 infection in pediatric patients seems to be clinically less severe than in adults, and it has a better prognosis. No evidence suggests a higher risk of COVID-19 infection in children with diabetes than in unaffected peers.	COVID-19 outbreak-modified T1D management and telemedicine have been demonstrated to be effective new tools for patient care.
Udsen et al. [9]	Department of Health Science and Technology, Aalborg University, Aalborg, Denmark	Systematic Review and Meta-analysis	22 studies were included (1615 participants).	To conduct a systematic review of the effectiveness of telemedicine solutions versus any comparator on diabetes-related outcomes among people with T1D.	The treatment effect for HbA1c% favoured telemedicine (mean difference of −0.26% [95% confidence interval: −0.37% to −0.15%]) with moderate effect certainty. Heterogeneity was moderate (I2 = 33.30%).	Reducing average HbA1c% levels is important to combat the risk of diabetic complications and premature death. However, the evidence mostly consists of small studies with relatively short durations, and the estimated pooled effect is smaller than could be expected from quality improvement strategies in general for diabetes management.
Iughetti et al. [47]	Pediatric Unit, Department of Medical and Surgical Sciences of the Mother, Children and Adults. University of Modena and Reggio Emilia, Modena, Italy.	Review	Search in PubMed. The terms used were COVID-19 OR coronavirus AND T1D, and as a filter, all articles about pediatric and adolescent populations were included.	To analyze why patients with T1D were poorly represented between the subjects hospitalized for COVID-19 and why the cases of diabetic ketoacidosis were fewer and more severe compared with the past years.	Telemedicine offers a way to be close to patients with T1D, even during the pandemic, as physical proximity is not always necessary.	Telemedicine was fundamental and probably simplified the relationship between the doctor and the patient, bringing them closer.
Southgate et al. [21]	Southampton Children’s Hospital, Southampton, United Kingdom	Scoping review	Searches in PubMed, Embase, and CINAHL databases on 15 April 2021 and 31 May 2022. Studies focused on telemedicine, remote consultation, video call, or remote patient monitoring in children (0–18 years) receiving outpatient care for diabetes, asthma, epilepsy, or renal disease.	To assess if telemedicine interventions are being evaluated in line with international WHO guidance as well as if there is sufficient evidence to support post-pandemic utilization of telemedicine in pediatric settings	In diabetes and asthma, conditions with established biomarkers that can objectively monitor disease progression, there were several studies with excellent confidence in the evidence.	There is promising evidence supporting telemedicine in pediatric settings. However, there is a lack of evaluation of telemedicine in comparison with usual outpatient care for noninferiority of clinical outcomes, and a more standardized approach to evaluate digital interventions is needed.
Huang et al. [48]	Fujian Medical University, Quanzhou, Fujian Province, China	Prospective randomized controlled study	92 eligible and randomized participants: 46 pt allocated to the intervention group and 46 pt in the control group.	To explore the effect of telehealth education and care guidance via WeChat on improving the quality of life of parents of children with T1D	6 months after discharge, the Self-Rating Anxiety Scale (SAS) and Self-Rating Depression Scale (SDS) scores of parents in the intervention group were significantly lower than those in the control group (*p* < 0.05). The scores of the physiological field, psychological field, social relationship field, and environmental field in the intervention group were higher than those of the control group according to the result of the WHO Quality of Life Brief Scale (WHOQOL-BREF; *p* < 0.05).	Health education and home care guidance via WeChat can effectively provide professional medical support and help to parents of children with T1D, which can effectively solve problems and improve their caring ability at home, relieve the pressure of home care, and improve their quality of life.
Rachmiel et al. [49]	Pediatric Endocrinology Unit, Shamir (Assaf Harofeh) Medical Center, Tzrifin, Israel	Observational multicentre real-life study	195 individuals with T1D (47.7% males), mean ± SD age 14.6 ± 5.3 years, and diabetes duration 6.0 ± 4.6 years. Telehealth was accomplished with 121 patients and their parents (62.0%); 74 (38.0%) did not transfer complete data.	To assess the feasibility and impact of telehealth visits on the time-in-range of pediatric individuals with T1D during the lockdown period	Improvement in glycaemic control by significantly less time spent in the severe hypoglycaemic range, less time spent in both hyperglycaemic and severe hyperglycaemic ranges, and more time spent in the range. Telehealth appears not to be a solution for all individuals. Personal tailoring of treatment is needed.	Pediatric and young adult patients with T1D benefited from a telehealth visit during COVID-19. However, this modality is not yet suitable for a considerable proportion of patients.
Bisno et al. [50]	Children’s Hospital Los Angeles, Los Angeles, CA, USA	Randomized control trial	Fifty-three YA (ages 18–25 years) with T1D participants of the Colorado Young Adults with T1D (CoYoT1) Clinic intervention, encompassing telehealth (TH) with or without VGA (virtual group appointments).	To analyze the impact of virtual group appointments (VGA) on self-reported health-related outcomes and care activities for young adults (YA) with T1D.	Participants in CoYoT1 Clinic reported significantly reduced diabetes distress compared to those in TH-only, who reported increased levels [Effect Size (ES) = −0.40, *p* = 0.02]. Participants in CoYoT1 Clinic reported improved self-management of T1D-related problem-solving (ES = 0.47, *p* = 0.051) and communication with care providers (ES = 0.39, *p* = 0.07).	Virtual group attendance in the CoYoT1 Clinic was associated with significant improvements in diabetes-related distress. Long-term exposure to VGA should be investigated in YA with T1D and other pediatric chronic conditions.
Bassi et al. [26]	Department of Neuroscience, Rehabilitation, Ophthalmology, Genetics, Maternal and Child Health, University of Genoa, Genoa, Italy	Observational study	290 patients with T1D (138 caregivers and 152 patients), mean age.	To evaluate the satisfaction in using telemedicine and telenursing of pediatric and young patients and their families followed by the Regional Pediatric Diabetes Center of Giannina Gaslini Institute, Genoa, Liguria, Italy.	The data collected showed that 92.4% of the population was overall satisfied with the quality of the service provided.	Telemedicine and telenursing have a positive impact on the daily lives of patients with type 1 diabetes and their parents. The most satisfied patients with the telemedicine service are pump users and residents furthest from the center.
Cobry et al. [51]	Barbara Davis Center for Diabetes, University of Colorado Anschutz Medical Campus, Aurora, Colorado, USA	Observational study	128 patients with T1D (baseline mean age 12.4–4.2 years) who utilized telemedicine were included.	To determine the use of diabetes devices in patients using telemedicine due to living far from the main diabetes specialty clinic.	Compared to the main clinic cohort (N = 3636), CGM and pump/CGM combination uses were lower among patients participating in clinic-to-clinic telemedicine (CGM: 29.7% vs. 56.0%, *p* < 0.001; CGM/pump combination: 27.3% vs. 40.3%, *p* = 0.004). Technology use was associated with lower A1c regardless of cohort.	Compared to patients attending in-person clinics, pediatric T1D patients who use clinic-to-clinic telemedicine due to their distance from the main clinic have lower CGM and combination CGM/pump use. For both telemedicine and main clinic patients, CGM and CGM/pump combination were associated with lower A1c.
Salabelle et al. [52]	Diabetes Department, Sud-Francilien Hospital, Corbeil-Essonnes, France	Observational study	77 patients with T1D diagnoses for more than one year. Mean age: 20.4 y.	To measure glucose control changes through the first French lockdown, focusing on telemedicine’s role.	Significant improvement in glucose control and self-monitoring in a population of adolescents and young adults with T1D over the lockdown period in France.	During lockdown, glucose control and self-monitoring improved in patients, with better results associated with telemedicine.
Chobot et al. [53]	Poland, Germany, Kuwait, Austria, USA, Canada, Italy, The Netherlands, India, Australia, Taiwan, Bangladesh, Slovenia	Observational multicentre study	66,985 youth with T1D from—82 responded, data available for 70 centres (42,798 youth with T1D).	To provide a global insight into initiatives in type 1 diabetes care driven by the COVID-19 pandemic and associations with glycemic outcomes.	Median HbA1c was lower in 2021 by 0.2% (1 mmol/mol) compared to 2018, *p* < 0.001. The insulin pump and CGM use significantly increased by 7.8% and 22.3% during the observational period, respectively (*p* < 0.001).	Changes to models of care delivery driven by the pandemic showed significant associations with HbA1c shortly after the pandemic outbreak and 2 years of follow-up. The association appeared independent of the concomitant increase in technology use among youth with T1D.
Zhang et al. [54]	School of Nursing, Fujian Medical University, Fuzhou, China	Systematic review and meta-analysis	20 RCTs (1704 participants from 12 countries) were included in the meta-analysis.	To systematically review the evidence on the effectiveness of telemedicine interventions compared with usual care on glycemic control among children and adolescents with T1D.	Telemedicine reduces HbA1c levels by 0.22 (95% CI –0.33 to –0.10; *p* < 0.001; I2 = 35%). Improvement in self-monitoring of blood glucose was not statistically significant. Telemedicine had no convincing effect on the Diabetes Quality of Life for Youth score satisfaction with diabetes, but there was a statistically significant improvement in non-youth-specific quality of life.	Telemedicine can reduce HbA1c levels and improve quality of life in children and adolescents with T1D. Telemedicine should be regarded as a useful supplement to usual care to control HbA1c levels and a potentially cost-effective mode. Meanwhile, researchers should develop higher-quality RCTs using large samples that focus on hard clinical outcomes, cost-effectiveness, and quality of life.
Umano et al. [55]	Department of Woman, Child, and of General and Specialized Surgery, University of Campania “Luigi Vanvitelli”, Naples 80138, Italy	Scoping review	Multiple studies investigated the use of telemedicine tools for the management of pediatric obesity and diabetes.	To review the current literature about the success of applying telemedicine as a healthcare service in support of pediatric obesity and diabetes.	Telemedicine is a concrete approach to ensuring the continuity of healthcare services for pediatric diabetes. It overcomes distance barriers and is cost-effective. However, new-onset cases need in-person healthcare assistance to ensure an adequate clinical evaluation of disease severity and family education in glucose monitoring and therapy.	Telemedicine is a powerful tool to address these issues, as it does not expose patients to contagion, it overcomes distance barriers, and it allows a multidisciplinary team approach.
Shah et al. [56]	University of Illinois College of Medicine, Chicago, IL, United States	Systematic review	11 studies were included; one of these was about telemedicine and type 1 diabetes.	To systematically evaluate the most recent evidence on the feasibility and accessibility of telemedicine services, patients’ and care providers’ satisfaction with these services, and treatment outcomes related to telemedicine service use among pediatric populations with different health conditions.	Telemedicine services for the public and pediatric care are comparable to or better than in-person services.	To maximize the potential of telemedicine, future research should focus on improving patients’ access to care, increasing the cost-effectiveness of telemedicine services, and eliminating barriers to telemedicine use.

CGM: Continuous Glucose Monitoring; CI: confidence interval; HbA1c: glycated hemoglobin; WHO: World Health Organization.

## Data Availability

No new data were created or analyzed in this study. Data sharing is not applicable to this article.
